# Altered Resting State Functional Activity and Microstructure of the White Matter in Migraine With Aura

**DOI:** 10.3389/fneur.2019.01039

**Published:** 2019-10-01

**Authors:** Péter Faragó, Eszter Tóth, Krisztián Kocsis, Bálint Kincses, Dániel Veréb, András Király, Bence Bozsik, János Tajti, Árpád Párdutz, Délia Szok, László Vécsei, Nikoletta Szabó, Zsigmond Tamás Kincses

**Affiliations:** ^1^Department of Neurology, Faculty of Medicine, Interdisciplinary Excellent Centre, University of Szeged, Szeged, Hungary; ^2^Central European Institute of Technology, Brno, Czechia; ^3^MTA-SZTE, Neuroscience Research Group, Szeged, Hungary; ^4^Department of Radiology, Faculty of Medicine, University of Szeged, Szeged, Hungary

**Keywords:** migraine, resting state, fMRI, white matter, diffusion, ALFF

## Abstract

**Introduction:** Brain structure and function were reported to be altered in migraine. Importantly our earlier results showed that white matter diffusion abnormalities and resting state functional activity were affected differently in the two subtypes of the disease, migraine with and without aura. Resting fluctuation of the BOLD signal in the white matter was reported recently. The question arising whether the white matter activity, that is strongly coupled with gray matter activity is also perturbed differentially in the two subtypes of the disease and if so, is it related to the microstructural alterations of the white matter.

**Methods:** Resting state fMRI, 60 directional DTI images and high-resolution T1 images were obtained from 51 migraine patients and 32 healthy volunteers. The images were pre-processed and the white matter was extracted. Independent component analysis was performed to obtain white matter functional networks. The differential expression of the white matter functional networks in the two subtypes of the disease was investigated with dual-regression approach. The Fourier spectrum of the resting fMRI fluctuations were compared between groups. Voxel-wise correlation was calculated between the resting state functional activity fluctuations and white matter microstructural measures.

**Results:** Three white matter networks were identified that were expressed differently in migraine with and without aura. Migraineurs with aura showed increased functional connectivity and amplitude of BOLD fluctuation. Fractional anisotropy and radial diffusivity showed strong correlation with the expression of the frontal white matter network in patients with aura.

**Discussion:** Our study is the first to describe changes in white matter resting state functional activity in migraine with aura, showing correlation with the underlying microstructure. Functional and structural differences between disease subtypes suggest at least partially different pathomechanism, which may necessitate handling of these subtypes as separate entities in further studies.

## Introduction

Migraine is the most widely known primary headache disorder, which affects nearly 10% of the population (1). The clinical appearance of attack is similar in most cases, however 20% of the patients report focal neurological symptoms (aura) before headache (migraine with aura; MWA). Different theories claimed to explain the pathomechanism of MWA and migraine without aura (MWoA) and the two forms of the headache may have distinct triggers (2). Malfunction of the trigemino-vascular complex, dysfunctional brainstem nuclei and altered metabolism of several neuropeptides may all play a part in the initiation of the migraineous headache. Aura symptoms are supposed to be originated from cortical spreading depression (CSD), a spreading wave of depolarization throughout the cortex that is thought to cause the focal symptoms associated with aura (3). CSD that is more prevalent in MWA is reportedly associated with the imbalance of excitation/inhibition in the migrainous brain (4). The altered excitability in MWA patients can cause increased amplitude of visual evoked potentials (5, 6), and also altered motion perception, where they have to judge certain properties of stimuli against a noisy background (7). Several studies also report enhanced responsiveness of the cortex to visual stimuli in a functional MRI setting (8, 9). Functional imaging placed the differences between the two subtypes on new foundations. Such methods were able to detect CSD during the aura phase (3, 10), and also revealed altered responses to sensory (11) or painful stimuli (12–14).

Resting state fMRI showed conflicting results about the alterations of brain activity during rest in interictal period. While some studies presented increased connectivity (15), some of them found the connectivity to be decreased (16). The fact, that these studies used heterogeneous populations of both MWA and MWoA patients, might explain this discrepancy. Our earlier investigation showed that the amplitude of the resting BOLD fluctuation, presumably a signature of increased excitability, was increased in MWA but not in MWoA (17).

Apart from gray matter functional networks, previous studies suggested that the white matter responds to external stimuli in a fashion similar to the gray matter, and this activation can also be measured with fMRI (18–20). Such fluctuations in activity are also present during rest, organized into distinct, consistently identifiable white matter resting state fMRI networks that bear close resemblance to gray matter functional networks (21–23). Several studies report that these networks function differently in neurological or psychiatric diseases (24–26). Since white matter microstructure is affected in migraine (27) and also differs between disease subtypes (28), a couple of questions arise. Does the functional activity of the white matter differs in disease comparing to the healthy population? Is there any difference between the various forms of migraine and if there it is, is it related to the microstructural alterations?

Resting state fMRI usually focus on the analysis of the whole frequency range of the time courses. However, it might be prudent to analyse different frequency ranges separately, since resting state networks were shown to be organize across several frequency bands (29). Several publications report resting state network abnormalities throughout several frequency bands in neuropsychiatric diseases and investigate the amplitude of low frequency fluctuations (30–33).

In our study, we aimed to explore differences in white matter resting state functional activity between the two migraine subtypes (MWA and MWoA) and healthy individuals. Considering the previously reported abnormalities of white matter microstructure, we also investigated whether these microstructural alterations are related to functional measures of the white matter during rest in migraine.

## Materials and Methods

### Participants

We recruited 51 migraine patients in our study (18 MWA, 33 MwoA). The participants belonged to the same cohort, similar to our previous investigation (17), all of them were enlisted from the Headache Outpatients Clinic of the Department of Neurology, University of Szeged. Apart from migraine, participants had no other neurologic or psychiatric disorders. All patients suffered from episodic migraine. None of the patients reported migraineous attack at in the preceeding week of the scanning. Each participant filled out a questionnaire about the prevalence and traits of the headache, medication and other diseases. None of the patient reported diabetes, or any other major cardiovascular diseases. Only one patient with aura was on interval therapy (iprazochrome). Seven of the 33 MWoA patients received interval treatment (1 topiramate, 1 amytriptilline, 5 iprazochrome).

As control group, we recruited 32 healthy volunteers, age and sex matched to the patients. Demographic data is included in Table 1.

**Table 1:** Demographic data of the participants.

	**MWA**	**MWoA**	**Controls**
*n*	18	33	32
Age (years; mean and SD)	32.1(8)	35.6(8.9)	35.2(11)
Gender (male)	3	3	2
Disease duration (years; mean and SD)	14.2(8.6)	13.7(9.1)	n.a.
Pain rated on visual analog scale	7.6(1.3)	8.7(1.2)	n.a.
Attack/year (days; mean and SD)	29(26)	55(45.6)	n.a.

The local ethics committee of the University of Szeged approved the study (authority No.: 87/2009). All participants gave written informed consent in accordance with the Declaration of Helsinki.

### Image Acquisition

MR imaging was performed on a 1.5 T GE Signa Excite HDxt MRI scanner. For every participant, we obtained high-resolution T1 weighted images (3D IR-FSPGR: TR/TE/TI: 10.3/4.2/450 ms, flip angle: 15°, ASSET: 2, FOV: 25^*^25 cm, matrix: 256^*^256, slice thickness: 1 mm,), a resting state fMRI protocol with echo-planar imaging technique (TE: 40 ms, TR: 3,000 ms, matrix: 64^*^64 cm, FOV: 30^*^30 cm, slice thickness: 6 mm, flip angle: 90°, NEX: 1, ASSET: 2,0 Ph, Phases per Loc: 128, volumes: 200) and 60 directions diffusion-weighted images with 6 non-diffusion-weighted reference volume [TE: 93.8 ms; TR: 16.000 ms; matrix: 96 * 96; FOV: 23 * 23 cm; flip angle: 90°; in-plane resolution: 2.4 * 2.4 mm; slice thickness: 2.4 mm; b: 1,000 s/mm^2^; number of excitations (NEX): 2; array spatial sensitivity encoding technique (ASSET) factor = 2]. Head motion was attenuated with foam pads.

### Data Processing

We performed data pre-processing and statistical analyses with FSL (FMRIB's Software Library) toolkits. The Fourier transformation and statistical correlation calculation was performed by Matlab.

### Pre-processing

We pre-processed resting state fMRI volumes using the FEAT toolbox, which included the removal of the first two non-steady-state volumes, removal of non-brain tissue via BET (34), motion correction (MCFLIRT), high pass filtering (cut-off sigma 100 s) and spatial smoothing with 6 mm FWHM. Because the motion parameters could alternate the BOLD signal, absolute and relative displacements were compared between groups. There were no significant differences in head movement. None of the subject's absolute displacement was larger then 0.8 mm.

All pre-processed fMRI images were registered to their own T1 images and to a standard structural image (MNI152, 2 mm isovoxel) with linear and non-linear registration respectively. To reduce calculation burden, we resampled all fMRI images to 4 mm isovoxel resolution. The pre-processed, standard space registered images were masked with a standard space white matter mask, thresholded at 0.99 probability.

We corrected diffusion data for eddy currents and movement artifacts by 12 degrees of freedom affine linear registration to the first non-diffusion-weighted reference image. Diffusion tensors at each voxel were fitted by FSL's FDT algorithm. Non-brain parts were removed with the brain extraction tool. We calculated fractional anisotropy (FA), mean diffusivity (MD), diffusivity axial (AD) and perpendicular (RD) to the principal diffusion direction for the whole brain, and aligned all subjects' FA images into a common space, using linear and non-linear registration. To match the resolution of functional volumes, we resampled the images to 4 mm isovoxel resolution.

### Extraction of White Matter Resting State Networks

We identified group level, spatially independent resting state fMRI networks using independent component analysis as implemented in FSL's MELODIC (35). We used whole study population (patients and controls together) for networks' identification. Standard space white matter images entered the analysis flow after voxel wise demeaning and variance normalization. The individual participants' 4D fMRI data were concatenated into one dataset and decomposed into a set of matrices describing spatial and temporal domains, so that they showed maximally non-Gaussian distribution (fast ICA algorithm). We set the number of independent components to 30 and thresholded component spatial maps at *p* < 0.5. We selected components for further evaluation based on the following criteria: (I) no activation outside the brain or in the gray matter, (II) temporal fluctuation and power spectrum characteristics common to resting state gray matter networks (36, 37), (III) a spatial pattern similar to the previously described white matter networks (21).

### Comparison of Networks and Power Spectrum Calculation

We used a dual regression approach for comparison between resting state white matter networks by using components' spatial maps as spatial regressors against the original subject-wise fMRI data, thus obtaining subject-specific versions of network time courses. We then regressed these time courses against the original fMRI data, which yielded individual spatial layouts for each network. To assess group differences in white matter network activity, we employed a standard GLM approach, with the group membership coded in the model. We used a non-parametric permutation test (5,000 permutations) for statistical inference, assessing cluster significance with the threshold-free cluster enhancement technique (38), and correcting for multiple comparisons via family wise error (FWE) correction (39).

To decompose network activity in the frequency domain, the time courses created in the first step underwent a Fast Fourier transform, generating a power spectrum for all individuals, for each network. We compared power spectra between groups by a two-sample *t*-test.

### Relationship Between Resting State Fluctuation and Diffusion Parameters

Voxel-wise correlation was calculated between the diffusion parameters and the group difference functional activity of the white matter. The analysis was restricted to the voxels showing significant group difference of functional activity at a threshold of *p* < 0.05. We used a non-parametric permutation test (5,000 permutations) for statistical inference, assessing cluster significance with the threshold-free cluster enhancement technique (38), and correcting for multiple comparisons via family wise error (FWE) correction (39).

### Correlation Between MRI Parameters and Clinical Data

Correlation was calculated between the diffusion parameters, functional activity of the white matter and clinical parameters. As dependent variable visual analog pain score (VAS), disease duration and attack frequency were used. MWA and MWoA groups were treated separately in our analysis. We used non-parametric permutation test for analysis and correcting for multiple comparisons via FWE.

## Results

### Demographical Data

Age and gender showed no significant differences between the three groups. The disease duration did not differ between the two migraine subgroups. MwoA patients showed significantly higher VAS of headache (*p* < 0.01) and attack frequency (*p* < 0.03) compared to MWA group.

### White Matter Resting State Functional Networks

Eight white matter networks were identified, according to our inclusion criteria and publication revealed previously (21). The networks' spatial distribution are shown in the Supplementary Figure S1. Three white matter networks showed significant difference between patients and controls:

- IC2 included the genu (rostrum) of the corpus callosum, fiber bundles extending bilaterally into the frontal poles.
- IC7 contained bundles in the body of the corpus callosum, fibers extending bilaterally into the frontal and parietal lobes.
- IC17 contained the bilateral occipital white matter.

### Different Expression of White Matter Functional Networks

#### MWA Compared to MWoA

The dual regression analysis identified higher expression in MWA compared to MWoA in all three networks.

**Figure 1:**
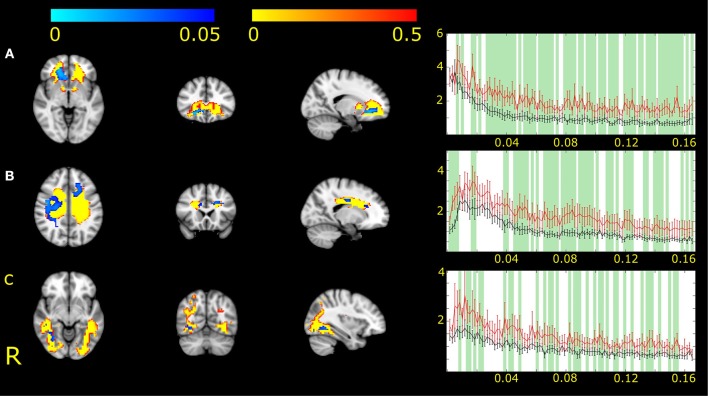
Three white matter functional networks (IC2, **A**; IC7, **B** and IC17, **C**) had different expression in MWA and MWoA. Higher expression and amplitude was found in the case of MWA. The white matter functional networks are depicted in red-to-yellow thresholded to 0.5 probability. The group differences between MWA and MWoA is shown in blue-to-light blue thresholded at *p* < 0.05. The colorbar represents *p*-values. The group average Fourier spectrum presented next to the spatial maps. The red line represents MWA, the black MWoA. Green columns showed frequency values that different significantly between the two groups (*p* < 0.05).

For the network representing the genu of the corpus callosum (IC2), we found differences between MWA and MWoA in the right hemisphere and also in midline (Figure 1A).

For the network including the white matter fibers crossing in the body of the corpus callosum (IC7) differences appeared in the left frontal anterior area and right parietal white matter (Figure 1B).

For the network including the bilateral occipital bundles (IC17) differences were found next to the medial side of the occipital gray matter (Figure 1C).

All areas with connectivity differences showed increased amplitude of fluctuation in MWA compared to MWoA.

#### MWA Compared to Healthy Volunteers

**Figure 2:**
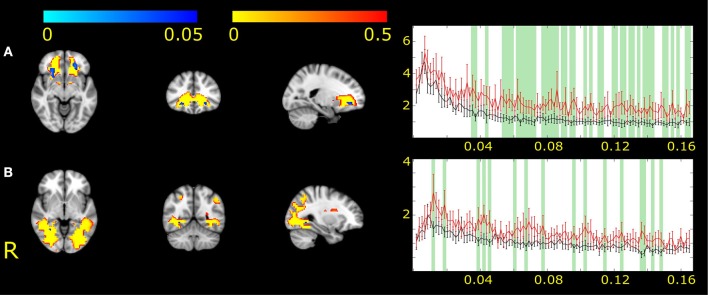
The two white matter functional networks showing differences between MWA and healthy volunteers (IC2, **A** and IC17, **B**). The white matter functional networks are depicted in red-to-yellow and thresholded to 0.5 probability. The group differences are shown in blue-to-light blue thresholded at *p* < 0.05. The colorbar represents *p*-values. The group average Fourier spectrum presented next to the spatial maps. The red line represents MWA, the black controls. Green columns showed frequency values that different significantly between the two groups (*p* < 0.05).

IC2 showed changes in the bilateral frontal white matter (Figure 2A). IC17 revealed changes in the right occipital pathways (Figure 2B). In the area of significance, the amplitude of fluctuation was higher in MWA patients compared to healthy volunteers.

#### MWoA Compared to Healthy Volunteers

There were no significant differences between healthy volunteers and MWoA patients in any of the white matter resting state networks.

### Connection Between Resting State Fluctuation and Underlying Microstructure

**Figure 3:**
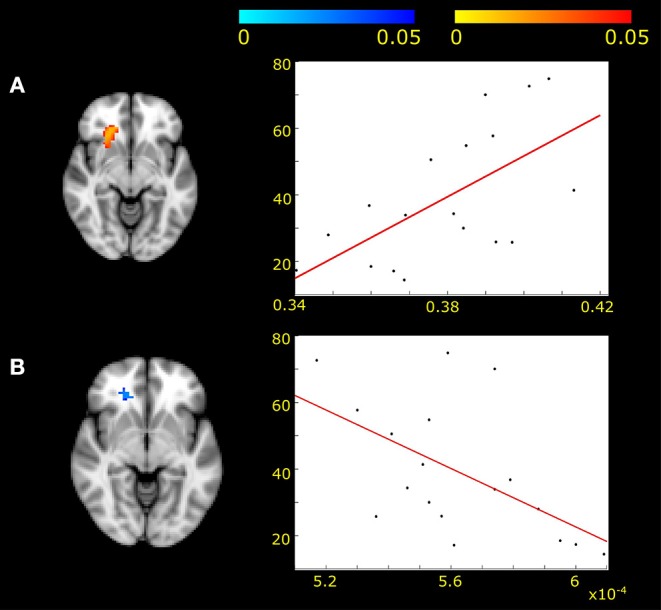
Positive correlation was found between fractional anisotropy and frontal white matter activity fluctuations **(A)** (*p* < 0.05, corrected for multiple correlations) in MWA patients. Negative correlation was found between radial diffusivity and frontal white matter activity in the same area **(B)** (*p* < 0.05). The x axis represents the diffusion values of FA **(A)** and RD **(B)**. The y axis shows the z-scores under the specified area.

The resting state fMRI fluctuation of the network including the genu of the corpus callosum (IC2) was correlated with measures of the underlying microstructure. We found positive correlation with FA (*r* = 0.61, *p* < 0.05) and negative correlation with RD (*r* = −0.55; *p* < 0.05) in MWA patients (Figure 3).

No other functional network activity was associated with diffusion parameters.

### Correlation Between MRI Parameters and Clinical Data

None of the resting state network, neither the diffusion parameters showed correlation with clinical parameters of the patients.

## Discussion

Our investigation showed that white matter functional networks similar to the gray matter resting state functional networks show alterations in migraine with aura. Three white matter networks showed stronger expression, primarily higher amplitude of activity fluctuation in MWA. Furthermore, alterations of resting state functional fluctuation showed a strong association with parameters of the underlying white matter microstructure, a feature that has not been reported before.

Conventional fMRI measures the BOLD signal in the gray matter. However, several studies found task dependent activation in the white matter as well (22, 40). A combined DTI and fMRI study found functional activation in a white matter tract connected to the cortex (41). Moreover, similar to the gray matter, the effect of anesthesia and hypercapnia is detectable in the BOLD response of the white matter (42, 43). There are, however, significant differences that separate WM activation from GM activation: for example, the power spectrum of the WM response is reduced (22), and the hemodynamic response function (HRF) also differs from that in the gray matter, as the WM response delayed and has a lower amplitude (44, 45). Furthermore, the WM HRF was more variable in separate tracts under functional loading (46). Although these previous studies used specialized fMRI sequences, it is widely accepted that the measured fMRI activation strongly depends on the HRF model. The different response may be attributed to lower vascular density or diameter (47, 48), or the lack of post synaptic potentiation in the white matter (49). Furthermore, the gray matter's relatively greater vascular volume, higher cerebral blood flow, metabolism and connected hemodynamic response (49, 50) results in a higher BOLD signal (41) compared to that of the WM.

It was also presented that BOLD activity fluctuation can be measured in the WM too. Resting state fMRI fluctuation in the WM showed a temporal profile similar to the GM, with a comparable power spectrum. There is a detectable connection between GM and WM tract activation in rest (22, 23, 37). Symmetrical white matter resting state networks can be identified, which show strong overlap with anatomical white matter tracts (21).

Previous electrophysiological and MRI studies found functional alterations in MWA patients compared to MWoA and healthy volunteers. Visual evoked potential studies registered increased response in MWA patients (5, 6). A TMS meta-analysis also found lower phosphene threshold in MWA, but not in MWoA (51). The background of such altered excitability might be the alteration of local neurotransmitter milieu. Lower GABA level an indicator of reduced inhibition (52), and higher glutamate/glutamine ratio was found in the MWA (53).

As an indirect fMRI evidence of the altered excitability we found increased amplitude of resting BOLD fluctuation in large scale GM networks in MWA compared to MWoA (17, 52). Our analysis showed that this dysfunctions extends into the white matter, with increased connectivity and higher amplitude of BOLD fluctuation. In the background of the white matter functional alterations can stand from the connected axonal function or axonal loading transport changes, which are consequences of altered gray matter activity. The increased axonal usage generates higher energy demand that may affect the callosal much more (54). Higher activity in the occipital fibers might stem from the altered occipital neuronal activity connected to CSD phenomenon (our MWA group of patients reported mostly visual aura).

In our earlier investigations we found white matter microstructural differences in migraine (27), mostly pronounced in MWA (28). Most importantly in the current study we found connection between microstructural and functional alterations. White matter resting state activity correlated strongly with FA and RD values of the corresponding white matter pathway, which are often interpreted as measures of myelin content and microstructural integrity (55). A recent review discusses a similar connection between fractional anisotropy and fMRI fluctuation (56), however, information about structure-function relationship in the white matter is scarce. A possible explanation for this connection is that increased myelin concentration requires an increased energy supply, which could translate to chronic BOLD fluctuation changes.

The signs of altered cortical excitability perceived not just in the gray, but also in the white matter in MWA. Being either the cause of or consequence, structural alterations can be found in migraine too, that is more pronounced in MWA. These results indicate fundamental differences in the two subtypes of the disease that needs attention in clinical care and also during development of therapeutic interventions.

Of course, out investigation is not without limitations. While the unequal size of the groups and the relatively small sample size are partially accounted for by the use of non-parametric statistical approaches these factors could potentially limit the generalisability of our findings. While theoretically one should account for multiple comparisons when investigating multiple ICs or functional networks, correction for multiple comparisons are potentially over-conservative and not frequently used in the literature.

## Data Availability Statement

The datasets generated for this study are available on request to the corresponding author.

## Ethics Statement

The studies involving human participants were reviewed and approved by the local ethics committee of the University of Szeged (authority No.: 87/2009). The patients/participants provided their written informed consent to participate in this study. Written informed consent was obtained from the individual(s) for the publication of any potentially identifiable images or data included in this article.

## Author Contributions

LV, ZK, NS, and PF planned the project and formulated the study hypothesis. ÁP, DS, and JT recruited the patients. ET, AK, BB, BK, DV, and KK organized and carried out the MRI measurements. BB, NS, and DV collected the clinical data. AK, PF, and DV analyzed the MRI data. DV, NS, ZK, PF, and JT formalized the discussion of the results. PF, ZK, NS, and AK wrote the manuscript.

### Conflict of Interest

The authors declare that the research was conducted in the absence of any commercial or financial relationships that could be construed as a potential conflict of interest.

## Supplementary Material

Supplementary Figure S1 Spatial distibuion of the identified white matter networks.
