# Supplementary figures and images for: Altered Resting State Functional Activity and Microstructure of the White Matter in Migraine With Aura
Source: Front Neurol. 2019 Oct 1;10:1039. doi: 10.3389/fneur.2019.01039 (PMC6779833; doi:10.3389/fneur.2019.01039)

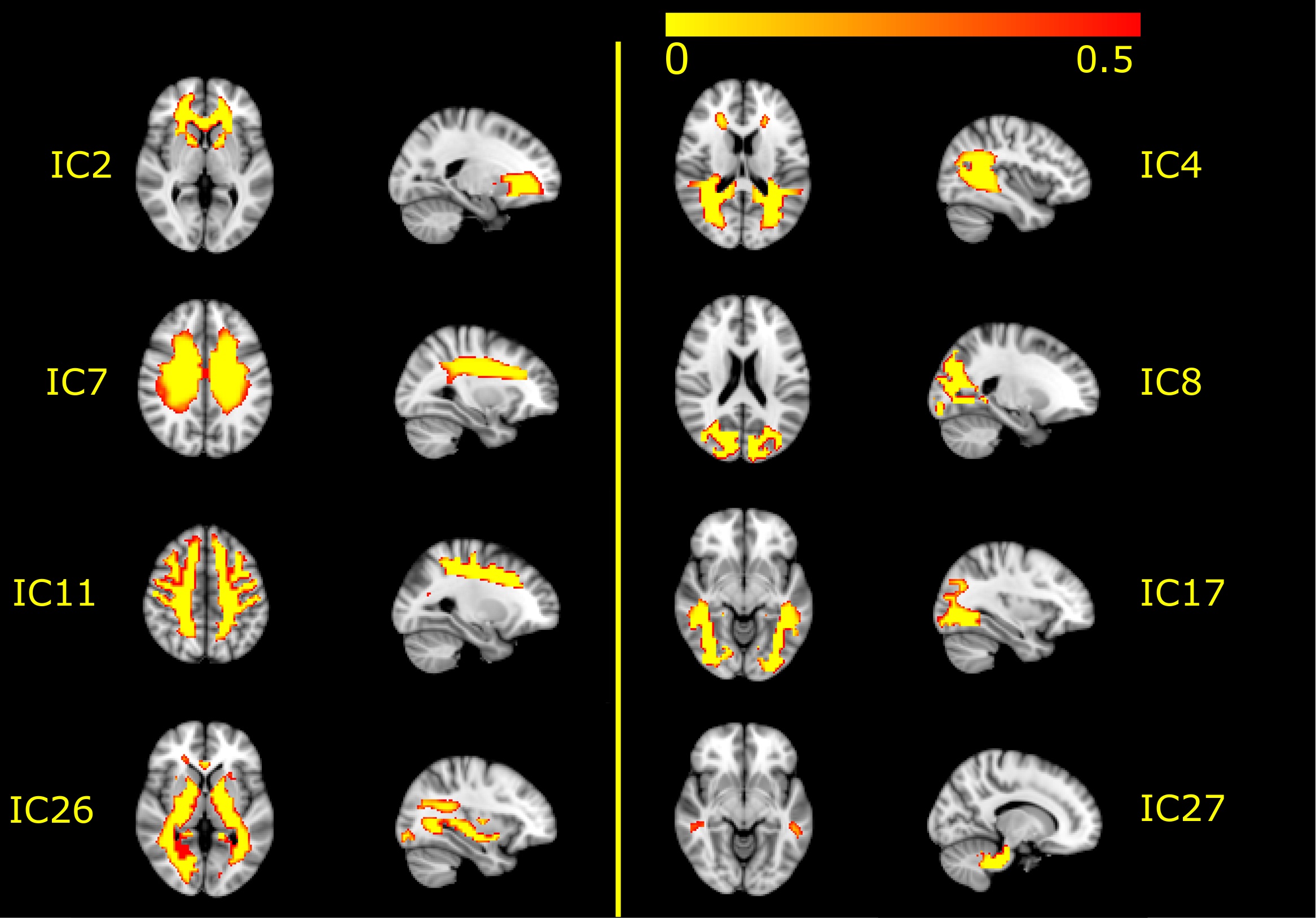

Supplement: Supplementary Figure S1 — Spatial distibuion of the identified white matter networks. [file Image_1.JPEG]
